# Supplementary material for: RalB directly triggers invasion downstream Ras by mobilizing the Wave complex
Source: eLife. 2018 Oct 15;7:e40474. doi: 10.7554/eLife.40474 (PMC6226288; doi:10.7554/eLife.40474)
Supplement: Supplementary file 1. [file elife-40474-supp1.docx]

**List of cell lines**

| Cell line | Reference | Generation Method | Selection Antibiotics |
| --- | --- | --- | --- |
| HEK-HT | Hanh et al, Nature 400, 464-468 (1999) |  | Hygromicin, Geneticin |
| HEK-HTRasV12 | Hanh et al, Nature 400, 464-468 (1999) |  | Hygromicin, Geneticin, Zeocin |
| HEK-HTRasV12G37 | Hanh et al, Nature 400, 464-468 (1999) |  | Hygromicin, Geneticin, Zeocin |
| OptoRal | This work | Lentiviral | Hygromicin, Geneticin |
| OptoControl | This work | Lentiviral | Hygromicin, Geneticin |
| OptoRal  iRFP-Abi1 WT | This work | Lentiviral | Hygromicin, Geneticin, Purmycin |
| OptoControl  iRFP-Abi1 WT | This work | Lentiviral | Hygromicin, Geneticin, Puromycin |
| OptoRal  iRFP-Abi1 Q56A | This work | Lentiviral | Hygromicin, Geneticin, Purmycin |
| MDA-MB-231 | ATCC (HTB-26) |  |  |
| BT549 | ATCC (HTB-122) |  |  |
| 293T | ATCC (CRL-3216) |  |  |
