# Supplementary material for: RalB directly triggers invasion downstream Ras by mobilizing the Wave complex
Source: eLife. 2018 Oct 15;7:e40474. doi: 10.7554/eLife.40474 (PMC6226288; doi:10.7554/eLife.40474)
Supplement: Supplementary file 2. [file elife-40474-supp2.docx]

**List of plasmids**

| Plasmids |
| --- |
| pHR-RalGEF-CRY2-mCherry (RalGEF domain = aa 1-518 of RGL2) |
| pHR-CRY2-mCherry |
| pLVX-CIBN-GFP-CAAX |
| pcDNA-Pak1GBD-iRFP (Pak1 aa 1-54) |
| pcDNA-Sec5GBD-iRFP (Sec5 aa 5-97) |
| pLVX-iRFP-Abi1 WT (Abi1 isoform6 [NM-001178119.1](https://www-ncbi-nlm-nih-gov.gate2.inist.fr/nuccore/NM_001178119.1)) |
| pLVX-iRFP-Abi1 Q56A |
| pLVX-GFP-RalB |
| pcDNA3-EGFP-Rac1 |
| pcDNA3-EGFP-Cdc42 |
