# Supplementary material for: RalB directly triggers invasion downstream Ras by mobilizing the Wave complex
Source: eLife. 2018 Oct 15;7:e40474. doi: 10.7554/eLife.40474 (PMC6226288; doi:10.7554/eLife.40474)
Supplement: Supplementary file 3.: [file elife-40474-supp3.docx]

**List of siRNAs**

| siRNA name | Target (sense) sequence |
| --- | --- |
| siCtrl | ON-TARGETplus Non-targeting siRNA #1 (Dharmacon) |
| siRalA-I | 5'-GAGACAACUACUUCCGAAG-3' |
| siRalA-IV | 5’-UCUUAAUCCUUUGGUGAAA-3 |
| siRalB-107 | 5'-UGACGAGUUUGUAGAAGAC-3' |
| siRalB-1749 | 5'-CAAAGACGUGAUGAGUUAA-3' |
| siRalB-1749 | 5'-CAGUAUCAUUGUUAAGUGA-3' |
| siRGL1-I | 5'-CCAUAAUACAGCUCCUAAA-3' |
| siRGL1-II | 5'-CCAUAAUACAGCUCCUAAA-3' |
| siRGL2- I | 5'-GCUAAUGUAUUCUACGCCA-3' |
| siRGL2-II | 5'-GGAUGGAGCUUCACACGAU-3' |
| siRGL3-I | 5'-ACACAGCCCUGCCGGAUAU-3' |
| siRGL3-II | 5'-GCGUCAGCAUCGACAAUGA-3' |
| siRalGDS-ft9 | 5'-AGCAAAUGCUAGACUUGAA-3' |
| siRalGDS-utr | 5'-AACCAGAGGACUAGCUGACUU-3' |
| siRalGPS1-ups1 | 5'-GAACAAAGAUCCAAUCAGA-3' |
| siRalGPS1-ups3 | 5'-GGAUAUACCUGUGUUUAAA-3' |
| siRalGPS2 #231 | 5'-GAUUCAGCAUACCCAUCAA-3' |
| siRalGPS2-ft10 | 5'-CAGUCGUUGGAGUUCUCAA-3' |
