# Supplementary material for: RalB directly triggers invasion downstream Ras by mobilizing the Wave complex
Source: eLife. 2018 Oct 15;7:e40474. doi: 10.7554/eLife.40474 (PMC6226288; doi:10.7554/eLife.40474)
Supplement: Supplementary file 4. [file elife-40474-supp4.docx]

**List of primers**

| Gene name | Sense | Antisense | Source |
| --- | --- | --- | --- |
| RGL1 | 5’-TGGTGATCAGGAATGCAATCG-3’ | 5’-CGGCATCATCCGTGTGAGATA -3’ | Sigma |
| RGL3 | 5’-CCTTGCAGAAGCACAATGTGC-3’ | 5’-CGTTGGCATTGTCAGGAATCA-3’ | Sigma |
| RalGDS | 5’-GGGGCTGAGTGAGGAGAAGC-3’ | 5’-GTGCTCCTTGCCCTTCTTGT-3’ | Sigma |
| RalGPS1 | Hs-RALGPS1-1-SG Cat no QT00054383 |  | Quiagen |

List Applied Biosystems probe sets (Taqman qPCR):

| Gene name | Probe set code |
| --- | --- |
| beta-2-microlobulin | Hs00984230-m1 |
| RGL2 | Hs00191084-m1 |
| RalGPS2 | Hs00216096-m1 |
